# Application of computed tomographic angiography and echocardiography in predicting left atrial appendage thrombosis in patients with non-valvular atrial fibrillation

**DOI:** 10.5830/CVJA-2022-052

**Published:** 2022-11-16

**Authors:** Xiaodan Wu, Fan Sun, Shoucheng Ma, Zhichen Wang, Shenghai Xu

**Affiliations:** Department of Ultrasound, Shenzhen University General Hospital, Shenzhen, China; Department of Ultrasound, Affiliated Hospital of Beihua University, Jilin, China; Department of Radiology, Jilin City TCM-WM Hospital, Jilin, China; Department of Cardiovascular Surgery, Jilin Central General Hospital, Jilin, China; Department of Ultrasound, Jilin Longtan District Tiedong Hospital, Jilin, China

**Keywords:** computed tomographic angiography, echocardiography, non-valvular atrial fibrillation, left atrial appendage, thrombosis

## Abstract

**Aim:**

:We aimed to explore the application of computed tomographic angiography (CTA) and echocardiography in predicting left atrial appendage (LAA) thrombosis in patients with non-valvular atrial fibrillation.

**Methods:**

The clinical data of 164 atrial fibrillation patients receiving cardiac CTA and real-time three-dimensional transoesophageal echocardiography (RT-3D-TEE) were retrospectively analysed. The patients were divided into group A (anticoagulant treatment group, n = 112) and group B (selective anticoagulant treatment group, n = 52) according to the CHA_2_DS_2_-VASc score, which scored for the presence or absence of congestive heart failure, hypertension, age ≥ 75 years, diabetes mellitus, stroke/transient ischaemic attack, vascular disease, age 65–74 years and gender (female). The CHA_2_DS_2_-VASc score was used to predict risk of thromboembolism from atrial fibrillation. The correlations of CHA_2_DS_2_-VASc score with CTA-based LAA classification and RT-3D-TEE measurement parameters were explored using Spearman’s analysis. Receiver operating characteristic (ROC) curves were plotted to explore the predictive value of CTA and RT-3D-TEE for LAA thrombus.

**Results:**

There were significant differences in age, disease course, hypertension, diabetes mellitus, coronary heart disease, heart failure, stroke/transient ischaemic attack/thromboembolism, vascular disease, B-type natriuretic peptide and serum uric acid levels, CHA_2_DS_2_-VASc score, LAA classification, left ventricular ejection fraction (LVEF), left atrial diameter (LAD), maximum diameter of LAA orifice, minimum diameter of LAA orifice and LAA length (p < 0.05). CHA2DS2- VASc score was positively correlated with cauliflower LAA, LAD, maximum diameter of LAA orifice, minimum diameter of LAA orifice and LAA length, and negatively correlated with LVEF (p < 0.001). ROC curve analysis indicated that CTA, RT-3D-TEE and CHA_2_DS_2_-VASc score had similar predictive values for risk of LAA thrombosis in atrial fibrillation patients, with the areas under the curve being 0.778, 0.814 and 0.792, respectively.

**Conclusion:**

Both CTA and RT-3D-TEE had high predictive values for LAA thrombosis in atrial fibrillation patients.

Non-valvular atrial fibrillation is a common type of arrhythmia that frequently occurs in elderly people.^1^ The atrial activation rate can be up to 300–600 beats/minute during an atrial fibrillation episode, leading to haemodynamic disorder in the cardiac cavity, and finally resulting in thrombus therein.^2^ Cerebral stroke or peripheral arterial embolism induced by atrial thrombus is the most harmful complication of atrial fibrillation.^3^

The left atrial appendage (LAA) is a high-incidence site of thrombosis in atrial fibrillation patients due to its special structure and function,^4^ therefore predicting LAA thrombosis in patients with atrial fibrillation is of significant importance for prevention and treatment of the disease. Presence or absence of congestive heart failure, hypertension, age ≥ 75 years, diabetes mellitus, stroke/transient ischaemic attack, vascular disease, age 65–74 years and female gender were scored with the CHA2DS2- VASc score and patients were placed into two groups for analysis.

The CHA_2_DS_2_-VASc score for thromboembolism risk from atrial fibrillation, a clinical tool commonly used to assess the risk of atrial fibrillation-induced cerebral stroke or thromboembolism, is able to guide risk stratification and prophylactic treatment, characterised by simple manipulation and easy implementation. However, it is rarely used for the cardiac structure.^5^

Computed tomographic angiography (CTA) can clearly display details of the blood vessels, heart and other structures, therefore it is often adopted to help understand the anatomical structure of the LAA before percutaneous LAA occlusion.^6^

As one of the routine examination items for atrial fibrillation patients, real-time three-dimensional transoesophageal echocardiography (RT-3D-TEE) can be employed to observe the morphology and structure of the LAA from different planes and angles and evaluate the functional status of the heart. It also serves as the gold standard for the diagnosis of LAA thrombus.^7^

In this study, we analysed the correlations of the CHA2DS2-

VASc score of 164 atrial fibrillation patients with CTA results and RT-3D-TEE measurement parameters. We investigated the application of CTA and RT-3D-TEE to predict LAA thrombosis in patients with non-valvular atrial fibrillation, so as to discover more feasible means for auxiliary examination of atrial fibrillation and related complications and improve the detection rate of LAA thrombus in such patients.

## Methods

A total of 164 atrial fibrillation patients undergoing cardiac CTA and RT-3D-TEE in our hospital from February 2015 to February 2020 were selected as the subjects of this study. There were 97 males and 67 females aged 49–81 years (64.34 ± 9.61). The inclusion criterion involved the patients definitely diagnosed with non-valvular atrial fibrillation by means of CTA and RT-3D-TEE.^8^ The exclusion criteria were set as follows: patients with valvular heart disease, those with a history of cardiac surgery, those with acute or subacute endocarditis, congenital cardiac structural abnormality, cardiac insufficiency, or those who could not undergo CTA.

This study was approved by the medical ethics committee of the hospital. All the patients and their families voluntarily agreed to participate in the study and signed the informed consent.

The CTA data were collected by experienced radiologists using Philips Brilliance 256-slice spiral-speed CT in strict accordance with the operation specifications. A contrast-medium hypersensitivity test was performed on the patients half an hour before scanning, and 12.5–25 mg of atenolol and 25–50 mg of metoprolol were orally administered after no adverse reactions had occurred. The patients were then instructed to control their heart rate and conduct breath-holding training.

The patients were scanned in the supine position, with sublingual administration of nitroglycerin tablets and connected to a high-pressure syringe and lead electrodes. A retrospective electrocardiographic gating technique was used for scanning in a single breath-hold at the end of exhalation, with a breathholding time of 10–15 seconds and a scanning range from the tracheal carina to the diaphragmatic surface of the heart.

Moreover, the descending aorta at the central level of the heart was monitored as a region of interest (threshold: 110–150 HU).

Subsequently, 60–80 ml of iopamidol (injection rate: 4–5 ml/s) were injected as a bolus into the cubital vein via a dual-tube highpressure syringe, and then 30–50 ml of normal saline were injected at a rate of 4.5–6 ml/s. Real-time contrast medium tracking and scanning were performed immediately, and coronary CTA scanning was executed automatically after reaching the threshold.

The scanning data were transmitted to an ADW4.6 postprocessing workstation, and two experienced radiologists were responsible for the post-processing of scanned images, including surface reconstruction, maximum-intensity projection, volume rendering and surface-shaded display. An LAA thrombus would be diagnosed if filling defects in the LAA were displayed after the injection of contrast medium and the density of the defect area was remarkably different from that of the surrounding tissues.

A Philips iE 33 colour Doppler ultrasonic diagnostic apparatus configured with MVQ analysis software was employed to complete RT-3D-TEE. A X7-2t multiplane probe, set at 2–7 MHz, was used for RT-3D-TEE and an X5-1 transthoracic probe was set at 1–5 MHz.

First, transthoracic echocardiography was conducted to examine the cardiac morphology, structure and function of patients. Specifically, the patients were deprived of food and water for four to six hours before examination and subjected to auxiliary anaesthesia with 2% lidocaine mortar. Then the patients were placed in the supine position, with the head tilted back, the mandible raised and the mouth biting the dental pad.

The X7-2t probe was smeared with an appropriate amount of coupling agent and the front end was inserted into the oesophagus, and 2D and colour Doppler ultrasonic scanning was implemented from varying depths, angles and sections when the probe reached the mid-oesophagus (30–40 cm away from the incisor teeth). Next, the probe direction was adjusted until the LAA was clearly and completely displayed, and the images were captured.

Thirdly, the sampling box was adjusted under the 3D imaging mode to clearly exhibit the LAA and peripheral cardiac structures, followed by collection of 3D images. Finally, the images were observed and analysed by two experienced sonographers. An LAA thrombus could be diagnosed if there were mass shadows in the LAA at discrete ultrasound planes, which had distinct edges and different densities from the surrounding tissues.

According to the CHA_2_DS_2_-VASc scoring standards,^9^ heart failure, hypertension, age ≥ 75 years, age = 65–74 years old, diabetes mellitus, stroke/transient ischaemic attack/thromboembolism, vascular disease and female gender were recorded as one, one, two, one, one, two, one and one point, respectively, with a total score of 10 points. It was recommended that the patients with a score ≥ two points were given anticoagulants for thrombus prevention and treatment, those with a score of one point were treated with selective anticoagulants or aspirin replacement therapy, and those with a score of zero points would not receive anticoagulants or merely use aspirin treatment.

The patients were assigned into group A (CHA_2_DS_2_-VASc score ≥ two points, anticoagulant treatment group, n = 112) and group B (CHA_2_DS_2_-VASc score < two points, selective anticoagulant treatment group, n = 52). Then we compared, between the two groups, clinical data such as age, gender, body mass index (BMI), disease course, paroxysmal atrial fibrillation, persistent atrial fibrillation, hypertension, diabetes mellitus, coronary heart disease, heart failure, stroke/transient ischaemic attack/thromboembolism, vascular disease, B-type natriuretic peptide, plasma fibrinogen and serum uric acid levels, CTA-based LAA classification, RT-3D-TEE measurement parameters [left ventricular ejection fraction (LVEF), left ventricular end-diastolic diameter (LVEDd), left ventricular end-systolic diameter (LVESd), left atrial diameter (LAD), LAA flow velocity (LAAV), maximum diameter of LAA orifice, minimum diameter of LAA orifice, LAA length and early diastolic peak velocity of mitral valve (E)] and CHA_2_DS_2_-VASc score.

## Statistical analysis

SPSS 21.0 software was utilised for statistical analysis. The normally distributed measurement data are expressed as mean ± standard deviation and the numerical data are represented as number and percentage. The t-test and chi-squared test were conducted for intergroup univariate analysis. The correlations of CHA_2_DS_2_-VASc score with CTA-based LAA classification and RT-3D-TEE measurement parameters were explored using Spearman’s analysis. Receiver operating characteristic (ROC) curves were plotted to explore the predictive values of CTA and RT-3D-TEE for LAA thrombus, and the area under the ROC curve (AUC), 95% confidence interval (CI), sensitivity and specificity were calculated; p < 0.05 suggested that the difference was statistically significant.

## Results

There were significant differences in the age, disease course, incidence of hypertension, diabetes mellitus, coronary heart disease, heart failure, stroke/transient ischaemic attack/ thromboembolism, vascular disease, B-type natriuretic peptide and serum uric acid levels and CHA_2_DS_2_-VASc score (p < 0.05), but the differences in gender, BMI, type of atrial fibrillation and plasma fibrinogen level were not significant between the two groups (p > 0.05) (Table 1).

**Table 1:** General data of patients

*Variables*	*Group A (n=112) =*	*Group B (n=52)*	*t/x2*	*p-value*
Age (years)	67.28 + 8.16	61.44 + 7.67	4.345	0.000
Gender, n (%)				
Male	70 (62.50)	31 (59.62)	0.125	0.724
Female	42 (37.50)	21 (40.38)		
BMI (kg/m²)	24.86 + 1.21	24.78 + 1.15	0.400	0.690
Disease course (month)	32.62 + 9.24	13.84 + 5.67	13.510	0.000
Type of atrial fibrillation, n (%)				
Paroxysmal	54 (48.21)	22 (42.31)	0.498	0.480
Persistent	58 (51.79)	30 (57.69)		
Hypertension, n (%)				
Yes	82 (73.21)	4 (26.92)	31.353	0.000
No	30 (26.79)	38 (73.08)		
Diabetes mellitus, n (%)				
Yes	29 (25.89)	(9.62)	5.726	0.017
No	83 (74.11)	47 (90.38)		
Coronary heart disease, n (%)				
Yes	37 (33.04)	7 (13.46)	6.931	0.008
No	75 (66.96)	45 (86.54)		
Heart failure, n (%)				
Yes	46 (41.07)	(3.85)	23.771	0.000
No	66 (58.93)	50 (96.15)		
Stroke/transient ischaemic attack/thromboembolism, n (%)				
Yes	28 (25.00)	0 (0.00)	15.676	0.000
No	84 (75.00)	52 (100.00)		
Vascular disease, n (%)				
Yes	(81.25)	(5.77)	82.701	0.000
No	21 (18.75)	49 (94.23)		
B-type natriuretic peptide (pg/ml)	329.45 + 132.77	138.49 + 59.16	9.912	0.000
Plasma fibrinogen (g/l)	2.81 + 0.36	2.71 + 0.51	1.442	0.151
Serum uric acid (umol/l)	414.17 + 45.73	362.08 + 56.27	6.298	0.000
CHA,DS,-VASc score	4.16 + 1.28	0.67 + 0.47	19.048	0.000
BMI, body mass index.				

**Fig. 1:**
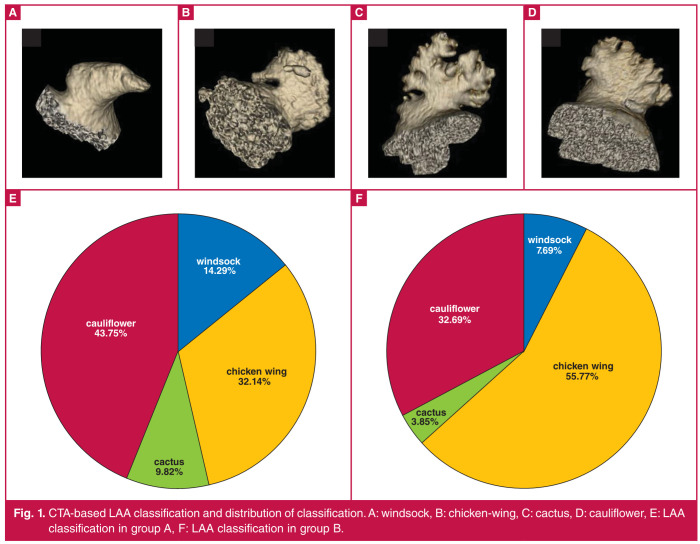
CTA-based LAA classification and distribution of classification. A: windsock, B: chicken-wing, C: cactus, D: cauliflower, E: LAA classification in group A, F: LAA classification in group B.

Group A had 16 cases of windsock LAA, 36 of chickenwing LAA, 11 of cactus LAA and 49 of cauliflower LAA. The numbers of cases of windsock LAA, chicken-wing LAA, cactus LAA and cauliflower LAA in group B were four, 29, two and 17, respectively. There were significant differences in LAA classification between the two groups (χ2 = 8.946, p = 0.030) (Fig. 1).

The LVEF, LAD, maximum and minimum diameter of the LAA orifice and LAA length showed significant differences (p < 0.05), while no significant differences in LVEDd, LVESd, LAAV and E were observed between the two groups (p > 0.05) (Table 2).

**Table 2:** RT-3D-TEE measurement parameters of patients

*Variables*	*Group A (n = 112)*	*Group B (n=52)*	*t*	*p-value*
LVEF (%)	62.01 + 6.03	64.19 + 5.98	2.160	0.032
LVEDd (mm)	45.02 + 3.87	46.31 + 5.40	1.743	0.083
LVESd (mm)	30.27 + 3.26	31.18 + 5.02	1.390	0.166
LAD (mm)	44.12 + 5.44	41.65 + 6.53	2.536	0.012
LAAV (m/s)	46.52 + 16.98	44.37 + 17.49	0.747	0.456
Maximum diameter of LAA orifice (mm)	21.47 + 4.46	19.35 + 4.72	2.781	0.006
Minimum diameter of LAA orifice (mm)	16.14 + 4.43	14.31 + 4.75	2.406	0.017
LAA length (mm)	32.04 + 5.39	28.45 + 4.76	4.114	0.000
E (cm/s)	0.98 + 0.19	1.01 + 0.18	0.956	0.340

LVEF, left ventricular ejection fraction; LVEDd, left ventricular end-diastolic

diameter; LVESd, left ventricular end-systolic diameter; LAD, left atrial diameter;

LAAV, LAA flow velocity; LAA, left atrial appendage; E, early diastolic

peak velocity of mitral valve.

The CHA_2_DS_2_-VASc score was positively correlated with cauliflower LAA (p < 0.001) but not with windsock, chicken-wing and cactus LAA (p > 0.05) (Table 3). The CHA_2_DS_2_-VASc score had a negative correlation with LVEF (p < 0.001), and it was positively correlated with LAD, maximum and minimum diameter of the LAA orifice and LAA length (p < 0.001) (Table 4).

**Table 3:** Correlations of CHA2DS2-VASc score with CTA results

*LAA classification*	*rs*	*p-value*
Windsock	0.017	0.186
Chicken-wing	0.064	0.214
Cactus	0.082	0.092
Cauliflower	0.424	< 0.001

**Table 4:** Correlations of CHA2DS2-VASc score with RT-3D-TEE measurement parameters

*RT-3D-TEE measurement parameter*	*rs*	*p-value*
LVEF	-0.341	< 0.001
LAD	0.220	< 0.001
Maximum diameter of LAA orifice	0.324	< 0.001
Minimum diameter of LAA orifice	0.305	< 0.001
LAA length	0.373	< 0.001

LVEF, left ventricular ejection fraction; LAD, left atrial diameter; LAA, left

atrial appendage.

**Fig. 2:**
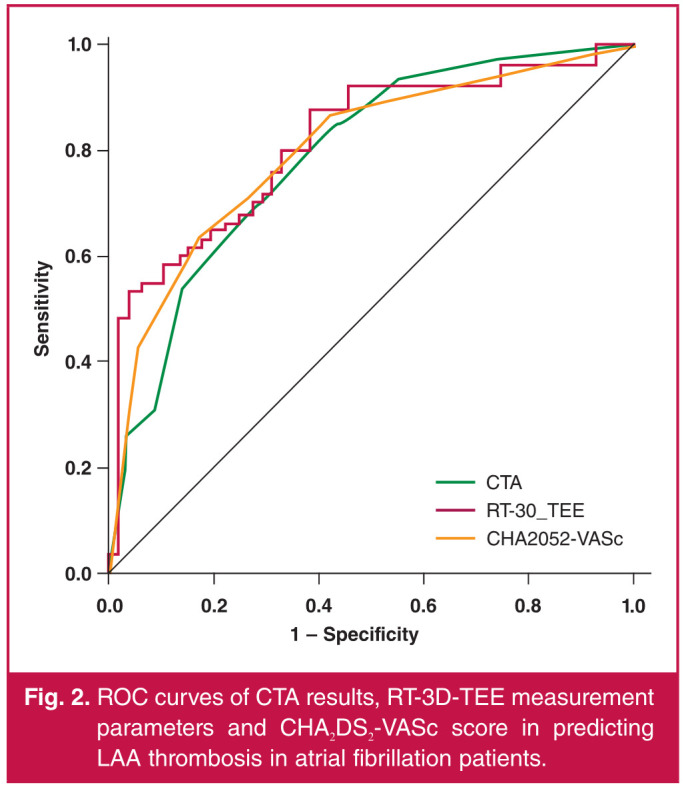
ROC curves of CTA results, RT-3D-TEE measurement parameters and CHA2DS2-VASc score in predicting LAA thrombosis in atrial fibrillation patients.

Fig. 2 shows ROC curves of the CTA results, RT-3DTEE measurement parameters and CHA_2_DS_2_-VASc score in predicting LAA thrombosis in atrial fibrillation patients. The predictive values of the CTA results, RT-3D-TEE measurement parameters and CHA_2_DS_2_-VASc score for LAA thrombosis in atrial fibrillation patients, analysed by ROC curves, are listed in Table 5. All the curves had large AUC and high sensitivity and specificity, suggesting that CTA results, RT-3DTEE measurement parameters and CHA_2_DS_2_-VASc score were significant for predicting LAA thrombosis in atrial fibrillation patients.

**Table 5:** Predictive values of CTA results, RT-3D-TEE measurement parameters and CHA2DS2-VASc score for LAA thrombosis in atrial fibrillation patients analysed by ROC curves

*Parameters*	*AUC*	*95% CI*	*Sensitivity (%)*	*Specificity (%)*	*Positive predictive value (%)*	*Negative predictive value (%)*
CTA	0.778	0.615-0.884	78.16	79.37	73.61	86.19
RT-3D-TEE	0.814	0.736-0.901	83.65	85.38	88.71	82.63
measurement parameter						
CHA,DS2 VASc	0.792	0.728-0.842	80.27	84.21	84.39	83.57

CTA, computed tomographic angiography; RT-3D-TEE, real-time three-dimensional transoesophageal echocardiography; AUC, area under the curve; CI, confidence interval; LAA, left atrial appendage.

## Discussion

As the Chinese society is aging, the number of atrial fibrillation patients and the overall prevalence rate of atrial fibrillation is rising annually. A study by Rashid and Layland demonstrated that the prevalence rate of atrial fibrillation was 0.71% among people aged above 35 years and as high as 2.35% among those aged above 75 years in China.^10^

Atrial fibrillation patients have weakened atrial contractability and blood stasis, so thrombus is easily formed in the atrium, causing cerebral stroke and other thromboembolic events. Clinically, the thrombus in approximately 90% of patients with non-valvular atrial fibrillation is generated from the LAA.^11^ The LAA is a residual accessory structure of the original left atrium in the embryonic period, with a blind pipe-shaped and long hooked structure, which has a different morphology among individuals and can be divided into windsock, chicken-wing, cactus and cauliflower types.^12^

The contractile and diastolic function of the LAA is involved in the filling and pressure regulation of the left ventricle, and the blood flow velocity is reduced by vortices easily formed in the LAA due to the abundant pectinate and trabecular muscles with rough surfaces.^13^ In the case of sinus rhythm, RT-3D-TEE can detect the clear-flow spectra for LAA emptying and filling.

For atrial fibrillation patients with a low-flow velocity, however, the left atrium and LAA need to expand the diameter and enhance active contraction to relieve the elevated pressure in the left atrium, thus decreasing the filling and emptying velocity of the LAA.^14^ Moreover, the LAA cannot be completely emptied because of the widened orifice and irregular inward motion of the atrial appendage wall, so the blood is blocked therein, thereby forming a thrombus. As a result, the LAA becomes a common site of atrial thrombus in atrial fibrillation patients.^15^

Among the RT-3D-TEE measurement parameters in the present study, the LVEF was lower, whereas the LAD, maximum and minimum diameter of the LAA orifice, and LAA length were larger in group A than in group B. The differential indices indicated that group A had more similar conditions to those of LAA thrombosis and a higher risk of thrombus.

According to several studies, the CHA_2_DS_2_-VASc score is a recognised, effective method for evaluating the risk of thromboembolism in atrial fibrillation patients and has been proven to be more accurate and valuable in predicting thromboembolism in patients with low-risk stroke.^16^,^17^ The study by Proietti et al.^18^ revealed that the survival curve of atrial fibrillation patients was correlated with the CHA_2_DS_2_-VASc score.

It was shown in this study that the CHA_2_DS_2_-VASc score was negatively associated with the RT-3D-TEE measurement parameter LVEF. However, it was positively correlated with LAD, maximum and minimum diameter of LAA orifice and LAA length.

Currently, the RT-3D-TEE technique is extensively applied in clinics and can automatically and clearly display the cardiac morphology and structure as well as quantify the cardiac function free of the impacts of rib and intra-pulmonary air, serving as a gold standard for detecting thrombus in the left atrium and LAA.^19^ Moreover, the analysis results of ROC curves denoted that the RT-3D-TEE measurement parameters and CHA_2_DS_2_-VASc score had similar predictive values, implying that RT-3D-TEE is of high value in predicting risk of LAA thrombosis in atrial fibrillation patients.

Although RT-3D-TEE is capable of directly exhibiting the morphology, structure and function of the left atrium and LAA of atrial fibrillation patients, it is characterised by difficulty in operating, certain requirements on doctors and an inability to perform on some patients. As a non-invasive imaging examination, CTA has a shorter scanning time, less radiation is used and simpler operation than RT-3D-TEE, so it is more acceptable. CTA images can not only clearly display the changes in cardiac blood flow and morphology of the left atrium and LAA through post-processing, but also precisely assess the structure and function of patient’s left atrium and LAA, thereby providing a reliable basis for clinical practice.^20^

Based on the correlations of CHA_2_DS_2_-VASc score with CTA-based LAA classification, the CHA_2_DS_2_-VASc score had a positive correlation with cauliflower LAA. Cauliflower LAA, the most complicated classification of LAA, has more trabecular muscles formed and lower-flow velocity than chicken-wing LAA.

Several studies have demonstrated that cauliflower LAA is an independent risk factor for cerebral stroke, transient ischaemic attack and LAA thrombosis.^21^,^22^ In this study, the analysis results of ROC curves revealed that the predictive value of CTA was close to that of the CHA_2_DS_2_-VASc score, suggesting that CTA possesses high value in predicting risk of LAA thrombosis in atrial fibrillation patients.

There were limitations in this study. For example, the time span of retrospective analysis was large, with certain selection and recall biases. The study data were obtained from a single source, and the sample size was small, so the results need to be further validated by multi-centre studies with larger sample sizes.

## Conclusion

Both CTA and RT-3D-TEE had fairly high predictive values for risk of LAA thrombosis in these atrial fibrillation patients. The CHA_2_DS_2_-VASc score could be applied to the clinical prediction of risk of LAA thrombus based on the actual condition of the patients. 
